# MAO-A Phenotype Effects Response Sensitivity and the Parietal Old/New Effect during Recognition Memory

**DOI:** 10.3389/fnhum.2018.00053

**Published:** 2018-02-13

**Authors:** Robert S. Ross, Andrew Smolen, Tim Curran, Erika Nyhus

**Affiliations:** ^1^Neuroscience and Behavior Program, Department of Psychology, University of New Hampshire, Durham, NH, United States; ^2^Institute for Behavioral Genetics, University of Colorado Boulder, Boulder, CO, United States; ^3^Department of Psychology and Neuroscience, University of Colorado Boulder, Boulder, CO, United States; ^4^Department of Psychology and Program in Neuroscience, Bowdoin College, Brunswick, ME, United States

**Keywords:** item memory, source memory, discriminability, EEG, ERP

## Abstract

A critical problem for developing personalized treatment plans for cognitive disruptions is the lack of understanding how individual differences influence cognition. Recognition memory is one cognitive ability that varies from person to person and that variation may be related to different genetic phenotypes. One gene that may impact recognition memory is the monoamine oxidase A gene (*MAO-A*), which influences the transcription rate of MAO-A. Examination of how *MAO-A* phenotypes impact behavioral and event-related potentials (ERPs) correlates of recognition memory may help explain individual differences in recognition memory performance. Therefore, the current study uses electroencephalography (EEG) in combination with genetic phenotyping of the *MAO-A* gene to determine how well-characterized ERP components of recognition memory, the early frontal old/new effect, left parietal old/new effect, late frontal old/new effect, and the late posterior negativity (LPN) are impacted by *MAO-A* phenotype during item and source memory. Our results show that individuals with the *MAO-A* phenotype leading to increased transcription have lower response sensitivity during both item and source memory. Additionally, during item memory the left parietal old/new effect is not present due to increased ERP amplitude for correct rejections. The results suggest that *MAO-A* phenotype changes EEG correlates of recognition memory and influences how well individuals differentiate between old and new items.

## Introduction

Personalized treatment for cognitive disruptions related to mental disorders relies on developing an understanding of individual differences in cognition. Genetic variation may be one component driving individual differences in cognitive ability (Friedman et al., 2008; Stelzel et al., 2010; Barnett et al., 2011; Markett et al., 2011; van Holstein et al., 2011; Colzato et al., 2013a,b; Ross et al., 2015). One gene that has been linked to symptoms of mental disorders, such as depression (Dannlowski et al., 2009) and anxiety (Tadic et al., 2003) as well as cognitive abilities, including episodic memory (Mueller et al., 2014), is the monoamine oxidase A gene (*MAO-A*). The *MAO-A* gene has a well characterized functional polymorphism that affects transcription of the MAO-A protein leading to changes in enzymatic activity (Sabol et al., 1998). The polymorphism is a variable number tandem repeat (VNTR) located in the promotor region of the *MAO-A* gene with homozygous female and hemizygous male carriers of the 4-repeat version of the gene (High MAO-A) showing increased transcription (Sabol et al., 1998). As MAO-A preferentially breaks down dopamine, serotonin and norepinephrine (Westlund et al., 1988; Willoughby et al., 1988; Saura et al., 1992), differences in MAO-A enzymatic activity may impact neurotransmitter function leading to individual differences in cognitive ability.

Genetic and pharmacological manipulation of MAO-A function in preclinical models, as well as different phenotypes of the *MAO-A* gene in humans, have been linked to differences in memory performance. Knocking out the *MAO-A* gene in mice leads to low levels of MAO-A activity, significant increases in serotonin and norepinephrine neurotransmitter levels (Cases et al., 1995), and increased fear conditioning (Kim et al., 1997; Singh et al., 2013). Rasagiline, an MAO-A and MAO-B inhibitor, and moclobemide, an MAO-A inhibitor, increase recognition and spatial memory in rodents (Steckler et al., 2001; Wong et al., 2010). Additionally, in a rat model of schizophrenia, the non-selective MAO inhibitor phenelzine increases memory performance (Simpson et al., 2012). Together, these preclinical studies suggest that low levels of MAO-A enzyme activity may be related to better recognition memory performance. However, in an examination of whether *MAO-A* phenotypes affect spatial memory in humans, Mueller et al. (2014) show that individuals with the *MAO-A* phenotype leading to higher MAO-A transcription (High MAO-A) perform better at spatial navigation in a virtual Morris water maze, a task used to assess episodic memory. The Mueller et al. (2014) study showing that high transcription of the *MAO-A* gene results in better memory is in direct contrast with the preclinical studies suggesting lower MAO-A activity improves memory (Kim et al., 1997; Wong et al., 2010; Simpson et al., 2012; Singh et al., 2013). Though these studies suggest that *MAO-A* phenotype may be related to individual differences in memory ability, it is unclear how *MAO-A* may affect memory. Examination of the underlying neural correlates of memory in combination with *MAO-A* genotyping may provide some clarity into how *MAO-A* phenotype impacts memory. Therefore, the current study uses electroencephalography (EEG) in combination with examination of *MAO-A* phenotype during performance of recognition memory tasks to determine how *MAO-A* may influence an individual’s memory performance.

Recognition memory tasks can be used to assess different aspects of long-term memory. Recognition memory can be assessed with item memory, where participants remember previously presented stimuli, and source memory, where participants remember a specific contextual detail associated with a previously presented stimulus. EEG research has characterized four distinct event-related potentials (ERPs) signals associated with recognition memory. There are three old/new effects, where the ERP amplitude is more positive for hits than correct rejections (Wilding and Rugg, 1996; Donaldson and Rugg, 1998, 1999; Rugg et al., 1998; Curran, 2000) and the late posterior negativity (LPN) where the ERP amplitude for hits has a larger negative change than correct rejections (Johansson and Mecklinger, 2003; Wilding et al., 2005; Mecklinger et al., 2007, 2016; Leynes and Phillips, 2008; Evans et al., 2010; Rosburg et al., 2011; Leynes and Kakadia, 2013). The first old/new effect is the FN400 which occurs 300–500 ms post-stimulus presentation over medial frontal scalp locations and is related to familiarity or conceptual priming (Mecklinger, 2006; Rugg and Curran, 2007; Voss et al., 2012). The second old/new effect is the left parietal old/new effect which occurs 500–800 ms post-stimulus presentation and has been linked to recollection (Wilding and Rugg, 1996; Donaldson and Rugg, 1998, 1999; Rugg et al., 1998; Curran, 2000; Curran and Hancock, 2007; Eichenbaum et al., 2007; Rugg and Curran, 2007). The third old/new effect is the late frontal old/new effect which occurs 1000–1500 ms post-stimulus presentation over right frontal scalp locations and is a signature of cognitive control processes (Rugg et al., 2003; Hayama et al., 2008; Hayama and Rugg, 2009). Finally, the LPN occurs over medial parietal scalp locations 1000–1500 ms post-stimulus presentation and may be associated with cognitive control demands during retrieval when task relevant contextual details are impoverished or overlap with other memory attributes (Johansson and Mecklinger, 2003; Leynes and Kakadia, 2013; Rosburg et al., 2013; Mecklinger et al., 2016). We compared these four ERP components of recognition memory in individuals with the High MAO-A and Low MAO-A genetic phenotypes. The advantage of using well characterized EEG measurements of recognition memory in combination with genetic phenotyping to examine individual differences is to illuminate how individuals may differ in performing recognition memory, even if accuracy and reaction time are the same. As each ERP component is believed to index different aspects of recognition memory task performance, determining which components may or may not be changed by *MAO-A* phenotype may reveal different strategies used to perform the memory tasks and may help resolve the conflicting findings for MAO-A activity levels from preclinical studies and human behavioral genetic studies. If memory processing is enhanced in the Low MAO-A group as suggested by the preclinical literature, then we would expect a larger FN400 and/or a larger left parietal old/new effect. However, if it is the High MAO-A group that leads to better memory as suggested by Mueller et al. (2014) results, we would see increased FN400 and/or parietal old/new effects in the High MAO-A group. Additionally, it may be that control processes are impacted by MAO-A phenotype, as such it may be that the late frontal old/new or LPN effects would be larger for the group with better recognition memory performance.

## Materials and Methods

### Participants

This study was carried out in accordance with the recommendations of the Institutional Review Board of the University of Colorado with written informed consent from all subjects in accordance with the Declaration of Helsinki. The protocol was approved by the Institutional Review Board of the University of Colorado. Participants for the study were recruited from the University of Colorado Boulder community. Data from these same participants examining how the 5HTTLPR serotonin transporter polymorphism affects ERP’s and how dopamine transporter polymorphisms affect ERP’s and oscillatory power during recognition memory has been reported elsewhere (Ross et al., 2015; Medrano et al., 2017). Participants were given monetary compensation for their participation. There were a total of 76 participants recruited for the study of which 59 participants, 33 males and 26 females, are included in the final data analysis (mean ± standard deviation = 20.7 ± 2.6 years old, with an age range of 18–29). The 17 participants removed from the study were due to multiple reasons. Four participants did not complete the EEG data recording sessions and three participants were removed due to technical reasons. Three participants were removed due to excessive blinking and two because of excessively noisy EEG channels. Four additional participants were removed due to low behavioral performance that when combined with epoch rejections resulted in less than 20 viable epochs per condition. One last participant was removed due to inability to classify MAO-A genetic phenotype into the High or Low MAO-A group (they had a 4R/5R phenotype). Thirty-seven participants (*n* = 13 females) were placed in the High MAO-A group, and 22 participants (*n* = 9 females) were placed in the Low MAO-A group.

### Stimuli

**Figure 1:**
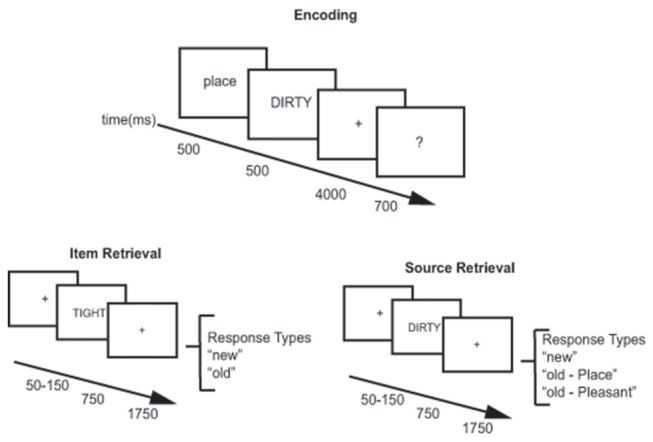
Behavioral paradigm. Encoding (top panel) for the item and source retrieval tasks used the same procedure. Participants were shown a 500 ms cue indicating which encoding task to use (pleasantness or place) followed by presentation of an adjective for 500 ms. They were given 4000 ms to perform the encoding task followed by a 700 ms question asking how well they felt they encoded the word. During item retrieval (bottom left) and source retrieval (bottom right), participants were shown a variable fixation cross (50–150 ms) followed by presentation of the adjective to be retrieved for 750 ms. A 1750 ms fixation cross followed adjective presentation. Participants could respond any time after the adjective was presented. During the item retrieval task responses were either old or new. During the source retrieval task responses were old place, old pleasant, or new.

The stimuli used for the experiment were comprised of 815 adjectives, 15 of which were used during practice sessions. The adjectives were words commonly used in the English language with a mean written word frequency of 34.86 according to the Kucera and Francis (1967) word norms. The average number of letters across the counterbalanced lists ranged from 6.87 to 7.00 letters per word and the average kfreq across counterbalanced lists ranged from 34.19 to 35.93. The kfreq and number of letters did not differ between lists. The words were presented to the participants in white uppercase letters in the center of the screen on a 26″ LCD computer screen with a black background at a visual angle of 2.3° using E-prime 2.0 (Psychology Software Tools Inc., Sharpsburg, PA, USA; Figure 1).

### Behavioral Task

Participants performed an item and source memory task separated by 2–5 days. During each visit, participants performed an encoding task. Following the encoding task, participants performed either an item memory or source memory task which was counterbalanced across participants. Participants came back to the laboratory 2–5 days later, performed the same encoding task with new stimuli, and performed the item or source memory task that they did not do on the first visit (i.e., if they did item memory on the first visit, they did source memory on the second visit). During the encoding task, participants encoded stimuli as they carried out a Place task and a Pleasantness task (Davachi et al., 2003; Kahn et al., 2004) with an equal number of words encoded in each task. The participants were given a 500 ms cue indicating which task, place or pleasant, they were to use with the next adjective. For the place task, participants were instructed to form a mental image of a spatial scene described by the adjective (for example, for “BEST,” their favorite eatery might be imagined). In the pleasantness task, the participants rated the pleasantness of the word. Before beginning the encoding task, a short practice of 10 study words was completed. After the practice block, participants began the encoding task, which involved presentation of 204 adjectives. Two words at the beginning and end of the list were included as primacy and recency buffers. Each adjective was presented for 500 ms. After adjective presentation, participants were given 4000 ms to perform the encoding task. A question mark was then presented for 700 ms, which instructed the participants to rate how successful they were at the source memory task on a 1–4 scale via button press. They could select 1 (unsuccessful), 2 (successful), 3 (with effort), or 4 (with ease).

Approximately 30 min after the completion of the encoding task, participants performed an item memory retrieval task on one visit and a source memory retrieval task on the other visit. EEG data was only collected during the retrieval portion of the tasks. Before beginning the item or source retrieval task, participants were presented with a short practice test block of 15 adjectives to ensure they understood test instructions. During the item memory task, participants were shown an adjective and asked to identify via button press if the word was old or new. They used the index finger of one hand for old and the index finger of the other hand for new. After their old/new response, participants indicated the confidence in their answer on a three-point scale, “surely”, “likely”, “maybe” using the index and middle finger of one hand and the index finger of the other hand. Adjectives were presented in blocks of 24 words, with two words at the beginning and end of the block serving as primacy and recency buffers. Twenty blocks were presented to each participant for a total of 200 old words, 200 new words and 80 buffer words. Each trial began with a 50–150 ms fixation period followed by a 750 ms adjective presentation period at which time participants were free to respond. Adjective presentation was followed by a 1750 ms fixation period. The source memory task was constructed the same way as the item memory task with 20 blocks of 24 adjectives for 200 old words, 200 new words and 80 buffer words. The difference was in the response options given to participants. For source memory, participants were instructed to indicate if the adjective was previously encoded with the place task, pleasantness task, or was a new word. Accuracy data and reaction time during the memory tasks was recorded using E-Prime 2.0.

### EEG Data Collection

A 128-channel HydroCel Geodesic Sensor NetTM connected to an AC-coupled, 128-channel, high-input impedance amplifier (200 MΩ, Net Amps TM, Electrical Geodesics Inc., Eugene, OR, USA) was used to collect scalp voltages from participants during the testing phase of the experiment. Individual sensors were adjusted until impedances were less than 50 kΩ, and amplified analog voltages (0.1–100 Hz bandpass) were digitized at 250 Hz.

### EEG Data Preprocessing

EEGLAB (Delorme and Makeig, 2004) and ERPLab (Lopez-Calderon and Luck, 2014) were used to pre-process EEG data. EEG channels were visually inspected for bad channels and interpolation was done using a spherical spline interpolation function (Srinivasan et al., 1996). If more than 4% of the channels needed interpolation (five channels), then that participant’s data was excluded from further analysis (*n* = 2). The data was filtered from 0.1 Hz to 40 Hz and re-referenced to the average signal. Then the data was epoched from 800 ms before stimulus presentation to 1500 ms after stimulus presentation (−800 ms to 1500 ms) for hit and correct rejection trials. A baseline of −800 ms to 0 ms was used for each epoch. After the data was split into epochs, participant data was put through a moving window artifact rejection where channel voltages registering a 100 mV change were rejected in 50 ms bins of 100 ms length. After artifact rejection, participants were required to have 20 artifact free epochs in each of the hit and correct rejection bins. Across participants, there was an average of 112.71 ± 38.13 (mean ± SD) hit trials and 106.07 ± 34.52 correct rejection trials in the item memory task and 113.48 ± 35.8 hit trials and 106.1 ± 38.86 correct rejection trials in the source memory task.

### Genotyping

Saliva samples were collected from subjects using a commercial product (Oragene™, DNAgenotek, Ottawa, ON, Canada) and allelic variants of the *MAO-A* gene were identified. The 4-repeat allelic variant of the *MAO-A* gene is the more efficient MAO-A enhancer, transcribing MAO-A 2–10 times more efficiently than other variations of the polymorphism (Sabol et al., 1998). Therefore, participants were grouped on the basis of the 4R variation. The *MAO-A* gene is located on the X chromosome, so males only have one allelic variant. Males with a 4R and females homozygous for the 4R variant (4R/4R) were placed in the High MAO-A group (*n* = 37). Males with a 3R variant and females heterozygous (3R/4R) or homozygous for the three repeat version (3R/3R) of the *MAO-A* gene were placed in the Low MAO-A group (*n* = 22). In females (*n* = 22), the genotypes were distributed according to Hardy-Weinberg equilibrium (59% 4R/4R, 27% 3R/4R, 14% 3R/3R). One participant was left out of the grouping as they had a 4R/5R allelic variant.

### Behavioral Analysis

Reaction times and proportion correctly identified were calculated for the item memory and source memory task conditions. In the item memory task, hits were defined as old items successfully identified as old while correct rejections were new items correctly identified as new. For the source memory task analysis, source hits were used, which were defined as stimuli where the correct source was identified. Using a significance of *p* < 0.05, 2 (hit vs. correct rejection) × 2 (High vs. Low MAO-A group) repeated measures analysis of variances (ANOVAs) were performed for item and source memory tasks independently, in order to determine any differences in reaction time and accuracy between genotype groups. Additionally, response sensitivity measured using *d*_a_, and response bias measures with *c*_a_ were compared between the High and Low MAO-A groups with independent samples *t*-tests.

### ERP ROI Analysis

**Figure 2:**
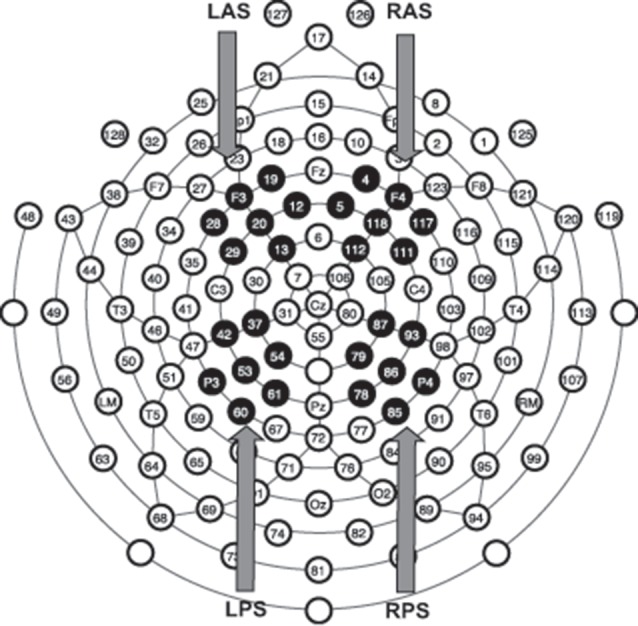
Regions of interest (ROIs) for event-related potentials (ERPs) analysis. Electrode montage representing the location of all 128 electrodes. Black filled electrodes represent the four different groups of seven electrodes averaged together to form the four ROIs for ERP analysis. LAS, left anterior superior; RAS, right anterior superior; LPS, left posterior superior; RPS, right posterior superior.

We analyzed the item and source data using groupings of seven electrodes for different scalp locations (regions of interest, ROIs) within specific timeframes (Figure 2), similar to our past approach (Norman et al., 2008; Nyhus and Curran, 2009; Ross et al., 2015). We examined four ROIs and three time frames due to their relevance to ERP effects related to recognition memory. The ROIs were the left anterior superior (LAS), the right anterior superior (RAS), the left posterior superior (LPS) and the right posterior superior (RPS). These labels were derived from prior recognition memory experiments (Curran, 2000; Curran et al., 2001; Ally and Budson, 2007; Ally et al., 2009; Depue et al., 2013) and are meant to describe electrode locations. The three time points analyzed were 300–500 ms, 500–800 ms and 1000–1500 ms post-stimulus presentation. The early old/new effect should appear in LAS/RAS 300–500 ms post-stimulus presentation while the LPS should demonstrate the left parietal old/new effect 500–800 ms post-stimulus presentation. The late frontal old/new effect should be seen in RAS 1000–1500 post-stimulus presentation while the LPN should be seen in LPS/RPS 1000–1500 post-stimulus presentation. We conducted four repeated measures ANOVAs to assess differences in mean ERP amplitudes using SPSS version 24 software (IBM Corporation, Armonk, NY, USA). For the early (300–500 ms) old/new effect, a 2 (condition; hits vs. correct rejections) × 2 (hemisphere; LAS vs. RAS) × 2 (group; High vs. Low MAO-A) repeated measures ANOVA was run. For the left parietal old/new effect (500–800 ms in LPS) a 2 × 2 repeated measures ANOVA was run with condition (hits and correct rejections) and group as variables. The third 2 × 2 ANOVA was for the late frontal old/new effect within RAS 1000–1500 ms and used condition and *MAO-A* group as factors. The final ANOVA was a 2 × 2 × 2 ANOVA assessing the LPN in the LPS and RPS 1000–1500 ms ROI using hemisphere, condition, and *MAO-A* group as factors.

## Results

### Behavioral Results

#### Item Memory

**Figure 3:**
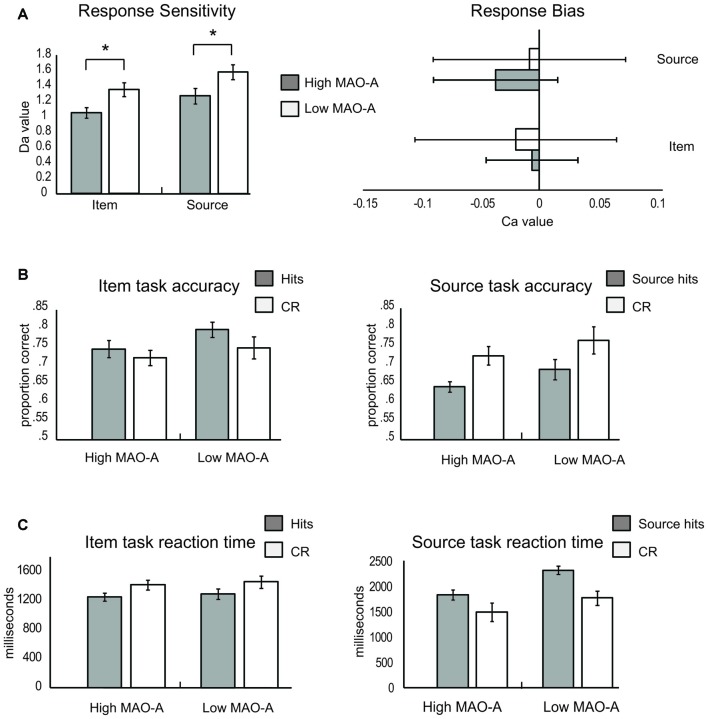
Item and source memory results. **(A)** Response sensitivity (left) and response bias (right) during the item and source memory tasks. High monoamine oxidase A (MAO-A) groups are represented by light gray bars and Low MAO-A groups by white bars. Response sensitivity (left panel) was lower in the High MAO-A groups during both item and source memory. There was no difference in response bias (right panel) between groups. **(B)** Item (left) and source (right) memory accuracy. The left graph illustrates the proportion of responses where a previously presented item was successfully identified as old (hits; gray) and where new items were successfully classified as new (correct rejections, CR, white) for High and Low MAO-A groups during item memory. No differences in accuracy were observed between groups during item memory. The right graph illustrates proportions correct during source memory. The gray bars represent the proportion of trials where source information was correctly identified (source hits). The white bars are the proportion of trials where new items were successfully classified as new (CR) for High and Low MAO-A groups during source memory. The proportion of source hits was significantly lower than correct rejections. **(C)** Item (left) and source (right) reaction time results. Hits were faster than CR across groups during item memory (left panel). During source memory, hits took longer than correct rejections (right panel). In addition, individuals with the High MAO-A phenotype were significantly faster during both hits and CR. *Indicates a significant difference at alpha level of 0.05.

The independent *t-*test examining response sensitivity (*d*_a_) during item memory revealed a significant difference (*t*_(57)_ = 2.4, *p* = 0.02, Cohen’s *d* = 0.71) between the High MAO-A (mean ± SEM; 1.05 ± 0.07) and Low MAO-A (1.35 ± 0.09; Figure 3A) groups. There were no significant differences in response bias (*c*_a_) between the two groups (Figure 3A). The 2 (hits vs. CR) × 2 (MAO-A group) repeated measures ANOVA examining proportion of correct responses during item memory revealed a non-significant main effect of condition (*F*_(1,57)_ = 1.76, *p* = 0.19, partial *η*^2^ = 0.03), a non-significant main effect of group (*F*_(1,57)_ = 3.04, *p* = 0.087, partial *η*^2^ = 0.051), and a non-significant condition × group interaction (*F*_(1,57)_ = 0.213, *p* = 0.646, partial *η*^2^ = 0.004; Figure 3B). Reaction time also failed to show a significant condition × group interaction (*F*_(1,57)_ = 0.005, *p* = 0.943, partial *η*^2^ < 0.001) or a main effect of group (*F*_(1,57)_ = 0.168, *p* = 0.683, partial *η*^2^ = 0.003). There was a significant main effect of condition (*F*_(1,57)_ = 40.415, *p* < 0.001, partial *η*^2^ = 0.415; Figure 3C), with hits being significantly faster than correct rejections.

#### Source Memory

Similar to item memory, the independent *t*-test examining response sensitivity (*d*_a_) during source memory revealed decreased response sensitivity in the High MAO-A (mean ± SEM = 1.27 ± 0.10) compared to the Low MAO-A group (1.58 ± 0.10; *t*_(57)_ = 2.05, *p* = 0.045, Cohen’s *d* = 0.57) with no difference in response bias (Figure 3A). The ANOVA examining proportion of correct responses during source memory revealed a significant main effect of condition (*F*_(1,57)_ = 15.42, *p* < 0.001, partial *η*^2^ = 0.21), where accuracy for correct rejections was better than for hits across groups. However, there was no main effect of group and no condition × group interaction (Figure 3B). Finally, there was a trend in source misattributions (*t*_(57)_ = 1.57, *p* = 0.087, Cohen’s *d* = 0.45), where those in the High-MAO-A group made more source misattributions (35.86 ± 1.4%) than the Low-MAO-A group (31.26 ± 2.5%). The examination of reaction time during source memory revealed a significant main effect of condition (*F*_(1,57)_ = 53.14, *p* < 0.001, partial *η*^2^ = 0.48), where hits took significantly longer than correct rejections. There was also a significant main effect of group (*F*_(1,57)_ = 5.63, *p* = 0.021, partial *η*^2^ = 0.09) with those in the High-MAO-A group being faster across both hits and correct rejections (Figure 3C). However, there was no condition × group interaction suggesting those in the High-MAO-A group were faster at making both hit and CR rejection judgments.

## ERP Results

### Item Memory

#### FN400

**Figure 4:**
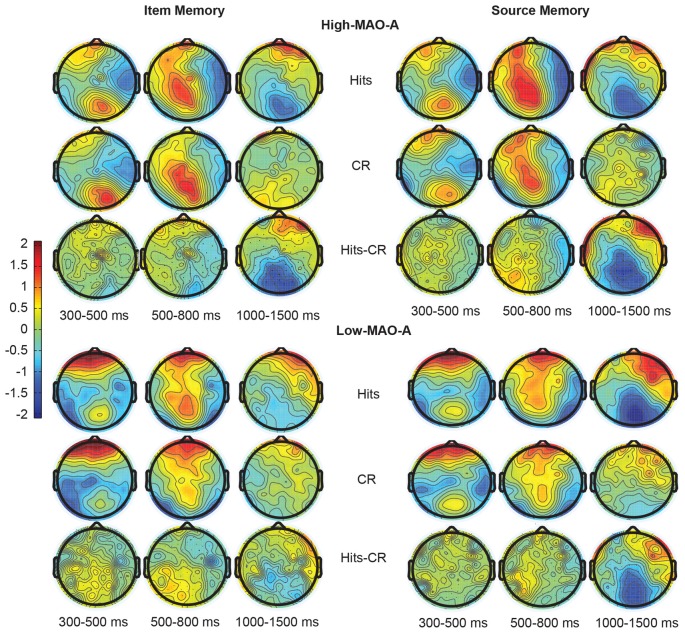
Topographic maps representing the distribution of ERP responses during item (left) and source (right) memory. The top two panels represent ERP responses in the High MAO-A group during Hits (top), correct rejections (CR; middle), and the difference between the hits and CR (bottom) 300–500 ms, 500–800 ms, and 1000–1500 ms post-stimulus presentation. The Low MAO-A group ERP responses are displayed in the bottom two panels with item memory on the left and source memory on the right. Hits for source memory are source hits where the source was correctly identified.

**Figure 5:**
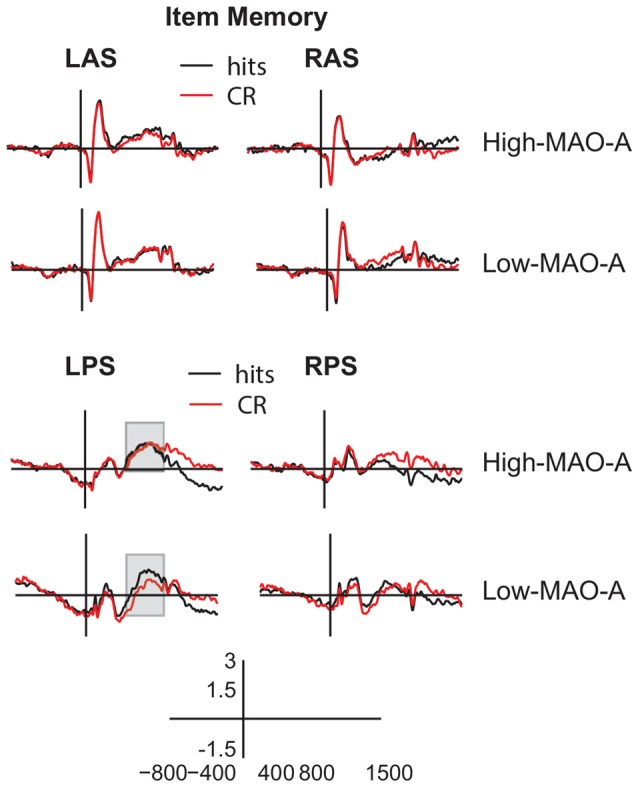
ERP waveforms during item memory retrieval. Averaged ERP waveforms from −800 ms to 1500 ms post-stimulus presentation (y axis crosses at 0 ms) in the left anterior superior (LAS, top left panels), (RAS, top right panels), left posterior superior (LPS, bottom left panels), and right posterior superior (RPS, bottom right panels) ROIs for hits (black) and CRs (red) during the item memory task. The gray boxes highlight the 500–800 ms timeframe in LPS where the High MAO-A and Low MAO-A groups showed significant differences in the parietal old/new effect.

The 2 (hemisphere) × 2 (hits vs. correct rejection) × 2 (MAO-A group) examining mean ERP amplitude in the LAS and RAS ROIs during item memory did not reveal any interaction between ERP amplitude and MAO-A genetic variant. The main effect of condition (partial *η*^2^ = 0.009), MAO-A group (partial *η*^2^ = 0.011), and the condition × MAO-A group interaction (partial *η*^2^ = 0.008) were not significant. There was a main effect of hemisphere (*F*_(1,57)_ = 6.97, *p* = 0.011, partial *η*^2^ = 0.11), with LAS showing significant higher mean amplitudes than RAS across both hits and correct rejections. No other interactions were significant (see Figure 4 for topographic maps and Figure 5 for ERP waveforms). These results suggest that the FN400 was not present across groups during item memory.

#### Parietal Old/New Effect

The 2 (condition) × 2 (MAO-A) group repeated measures ANOVA examining mean ERP amplitude in the LPS ROI 500–800 ms post-stimulus presentation revealed a significant main effect of condition (*F*_(1,57)_ = 4.89, *p* = 0.031, partial *η*^2^ = 0.08) and a significant condition × MAO-A group interaction (*F*_(1,57)_ = 4.20, *p* = 0.045, partial *η*^2^ = 0.069). The main effect of group was not significant (*F*_(1,57)_ = 1.51, *p* = 0.224, partial *η*^2^ = 0.03). A *post hoc* paired samples *t-test* comparing mean hit (0.85 ± 0.26 μV) to correct rejection (0.33 ± 0.28 μV) amplitude within the MAO-A Low group showed a significant difference (*t*_(21)_ = 3.28, *p* = 0.004, Cohen’s *d* = 0.41), revealing the presence of the parietal old/new effect. Critically, there was no significant difference between mean hit (1.11 ± 0.31 μV) and correct rejection (1.09 ± 0.28 μV) amplitudes in the MAO-A High group (*t*_(36)_ = 0.133, *p* = 0.90, Cohen’s *d* = 0.01) suggesting the parietal old/new effect is not present in individuals with high MAO-A transcription efficiency (Figures 4, 5). *Post hoc* independent *t*-tests comparing mean hit and CR amplitudes across groups showed no difference in mean hit amplitude between groups (*t*_(57)_ = 0.59, *p* = 0.56, Cohen’s *d* = 0.16) and marginal difference in mean CR amplitude (*t*_(57)_ = 1.8, *p* = 0.07, Cohen’s *d* = 0.50) between groups. These results suggest that the lack of a parietal old/new effect in the High MAO-A group may be due to an increase in mean CR amplitude.

#### Late Frontal Old/New Effect

The 2 (condition) × 2 (MAO-A group) ANOVA examining mean ERP amplitudes in RAS 1000–1500 ms post-stimulus presentation revealed a significant main effect of condition (*F*_(1,57)_ = 6.59, *p* = 0.013, partial *η*^2^ = 0.104) with mean hit amplitude across groups being higher than mean CR amplitude. There was no main effect of group (partial *η*^2^ = 0.03) and no significant condition × MAO-A group interaction (partial *η*^2^ = 0.008; Figures 4, 5).

#### LPN

Differences in mean ERP amplitudes in the LPN across MAO-A groups were examined with a 2 (condition) × 2 (hemisphere) × 2 (MAO-A group) repeated measures ANOVA in the LPS and RPS ROIs 1000–1500 ms post-stimulus presentation. The ANOVA revealed a significant main effect of condition (*F*_(1,57)_ = 22.52, *p* < 0.001, partial *η*^2^ = 0.28) with mean hit amplitude being significantly lower than mean correct rejection amplitude (Figures 4, 5). The significant main effect of condition suggests the presence of the LPN in both groups. However, there were no significant interactions with MAO-A group nor was there a main effect of MAO-A group suggesting that the LPN during item memory is not affected by MAO-A transcription efficiency.

### Source Memory

#### FN400

**Figure 6:**
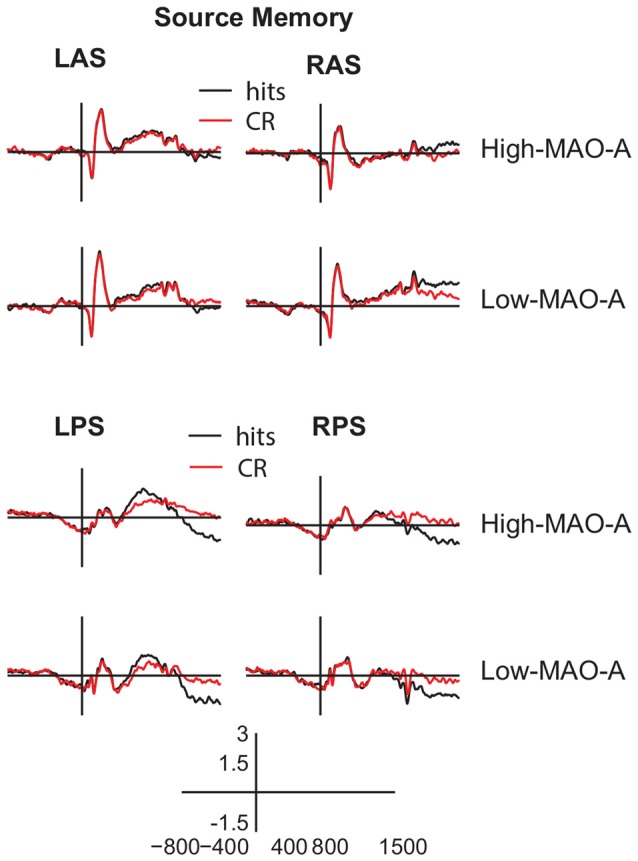
ERP waveforms during source memory retrieval. Averaged ERP waveforms from −800 ms to 1500 ms post-stimulus presentation (y axis crosses at 0 ms) in the (LAS, top left panels), (RAS, top right panels), (LPS, bottom left panels), and (RPS, bottom right panels) ROIs for hits (black) and CRs (red) during the source memory task. The gray box represents the main effect of group for the late frontal old/new effect where ERP amplitudes in the Low MAO-A group were higher than the High MAO-A group. However, there were no significant condition by group interactions during source memory.

The 2 (hemisphere) × 2 (hits vs. CR) × 2 (MAO-A group) examining mean ERP amplitude in the LAS and RAS ROIs revealed a significant main effect of condition (*F*_(1,57)_ = 6.51, *p* < 0.01, partial *η*^2^ = 0.10) with mean hit amplitude being greater than mean correct rejection amplitude (Figures 4, 6). The difference in hit and CR amplitude suggests the FN400 was present during source memory. There was also a main effect of hemisphere (*F*_(1,57)_ = 6.03, *p* = 0.017, partial *η*^2^ = 0.1) where the left hemisphere had higher ERP amplitudes. However, there were no significant interactions with MAO-A group nor was there a main effect of MAO-A group suggesting that the FN400 during source memory is not affected by MAO-A transcription efficiency.

#### Parietal Old/New Effect

The 2 (hits vs. CR) × 2 (MAO-A group) ANOVA comparing mean ERP amplitudes in the LPS ROI 500–800 ms post-stimulus presentation revealed a main effect of condition (*F*_(1,57)_ = 10.45, *p* < 0.01, partial *η*^2^ = 0.16; Figures 4, 6). Mean hit amplitudes were larger than mean CR amplitudes revealing the presence of the left parietal old/new effect across groups. Because there was a difference in the parietal old/new effect during item memory, we ran *post hoc* tests to verify that the parietal old/new effect during source memory was present in both groups. The *post hoc* paired samples *t*-tests revealed the presence of the parietal old/new effect in both the High MAO-A (*t*_(36)_ = 2.649, *p* = 0.012, Cohen’s *d* = 0.44) and Low MAO-A groups (*t*_(21)_ = 2.496, *p* = 0.02, Cohen’s *d* = 0.53). However, there was no main effect of group or a condition × group interaction suggesting that MAO-A phenotype does not influence the left parietal old/new effect during source memory.

#### Late Frontal Old/New Effect

The 2 (condition) × 2 (MAO-A group) ANOVA comparing mean ERP amplitudes 1000–1500 ms post-stimulus presentation in the RAS ROI revealed a significant main effect of condition (*F*_(1,57)_ = 11.27, *p* < 0.01, partial *η*^2^ = 0.17). There was also a main effect of group (*F*_(1,57)_ = 7.36, *p* < 0.01, partial *η*^2^ = 0.11) where mean ERP amplitudes in the Low MAO-A group (0.91 μV ± 0.21) were higher than in the High-MAO-A group (0.20 μV ± 0.16). However, there was no condition × MAO-A group interaction (*F*_(1,57)_ = 0.066, *p* = 0.799, partial *η*^2^ = 0.001; Figures 4, 6). These results suggest that the late frontal old/new effect is present across groups and that activity under right frontal scalp locations is higher in the Low-MAO-A group during source memory.

#### LPN

The 2 (hits vs. CR) × 2 (hemisphere) × 2 (MAO-A group) ANOVA comparing mean ERP amplitudes in the LPS and RPS ROI’s 1000–1500 ms post-stimulus presentation revealed a significant main effect of condition (*F*_(1,57)_ = 26.382, *p* < 0.001, partial *η*^2^ = 0.32) with hits significantly more negative than CR (Figures 4, 6). There were no other significant main effects or interactions. These results suggest the LPN was present across MAO-A group and that MAO-A phenotype does not affect the LPN during source memory.

## Discussion

The present study investigated how different phenotypes of the *MAO-A* gene change ERP correlates of recognition memory in order to explore how *MAO-A* genetic variation contributes to individual differences in recognition memory. We show that MAO-A phenotype changes behavioral performance as well as EEG correlates of recognition memory. During item memory, individuals in the High MAO-A group, which includes women homozygous for the 4R phenotype and men hemizygous for the 4R phenotype, have lower response sensitivity scores (Figure 3A). Additionally, the left parietal old/new effect is not present in High MAO-A individuals during item memory due to an increase in mean correct rejection amplitude (Figure 5). During source memory, the High MAO-A group show lower response sensitivity scores and faster reaction times for both hits and correct rejections (Figure 3C). They also showed an overall decrease in mean hit and CR amplitude for the late frontal old/new effect. The pattern of behavioral and ERP findings show that MAO-A phenotype changes the left parietal old/new effect and influences an individual’s ability to differentiate old from new items during recognition memory.

### Increased Noise during Item Memory in High MAO-A Individuals

MOA-A levels may influence the ability to distinguish between old and new items. Response sensitivity directly measures the ability to discriminate between old and new stimuli. During both item and source recognition memory, the High MAO-A group have lower response sensitivity scores (Figure 3A). We also show that the left parietal old/new effect is absent in the High MAO-A group during item memory due to an increase in mean ERP amplitude for correct rejections (Figures 4, 5). As the mean amplitudes for correct rejections is increased to the same level as hits in the High MAO-A group, we suggest the left parietal ERP in the High MAO-A group is signaling new stimuli as if they were old stimuli, resulting in more noise. The increased noise may then be reflected behaviorally by a decrease in response sensitivity. The individuals in the High MAO-A group possess the phenotype leading to increased transcription of MAO-A (Sabol et al., 1998), and presumably faster clearance of monoamines due to increased MOA-A enzymatic activity. Combined with our results showing the lack of a parietal old/new effect and decreased response sensitivity in the High MAO-A group, these results suggest that different MAO-A levels resulting from *MAO-A* phenotype changes an individual’s ability to differentiate old and new stimuli.

Our results may help explain why preclinical models show that knocking out (Kim et al., 1997; Singh et al., 2013) or blocking MAO-A activity (Steckler et al., 2001; Wong et al., 2010) leads to better memory. Knocking out or blocking MAO-A activity leads to lessened MAO-A enzymatic activity, which is akin to the Low MAO-A group in our study. The Low MAO-A group had higher response sensitivity scores (Figure 3). Additionally, our results show that the left parietal old/new effect is present in the Low MAO-A group and absent in the High MAO-A group due to an increase in mean ERP amplitude for correct rejections (Figures 4, 5). In preclinical models, decreasing the activity of MAO-A may make it easier to distinguish between old and new stimuli leading to the better memory seen in preclinical knockout and pharmacological manipulation studies.

### Source Memory Differences in High MAO-A Individuals

The individuals in the High MAO-A group may be responding more carelessly with less cognitive control during source memory. The High MAO-A group showed faster reaction times for both hits and correct rejections during source memory. Additionally, our results show a decrease in overall ERP amplitude for both hits and CRs for the late frontal old/new effect, which is an index of cognitive control (Wilding and Rugg, 1996; Donaldson and Rugg, 1998; Ranganath and Paller, 2000; Werkle-Bergner et al., 2005; Hayama et al., 2008; Hayama and Rugg, 2009). Though the late frontal old/new effect is still present, the decreased amplitudes may indicate less overall cognitive control. Importantly, individuals with the High MAO-A phenotype have less attention control compared to Low MAO-A individuals (Fossella et al., 2002), supporting our interpretation of decreased cognitive control ability in the High MAO-A group. More direct evidence of decreased cognitive control in High MAO-A individuals comes from a study examining working memory updating. Enge et al. (2011) show decreased N2 ERP amplitude for the High MAO-A group during the n-back task. The N2 ERP is associated with increased cognitive control (Folstein and Van Petten, 2008; Enge et al., 2011; Pires et al., 2014; Kropotov et al., 2017) suggesting the High MAO-A group does not engage cognitive control during the n-back task as strongly as Low MAO-A individuals. When combined with the faster RTs and decreased late frontal old/new effect seen in our study, these results may suggest the High MAO-A group engages less control during source memory.

An alternative interpretation to our suggestion that the High MAO-A group may engage in less cognitive control during source memory is that the High MAO-A group may process information more efficiently. The Low MAO-A phenotype has been linked to increased impulsivity (Verdejo-García et al., 2013) in addiction. Additionally, a recent behavioral study examining the effect of *MAO-A* phenotype on spatial memory using a virtual water-maze in humans suggests that the High *MAO-A* phenotype leads to more efficient navigation performance (Mueller et al., 2014). Specifically, men in the High MAO-A group had shorter path lengths and less heading error than those in the Low MAO-A group, though there was no difference in the time it took to get to the escape platform. Our results do show that the High MAO-A group was faster at responding during the source memory task and other studies also show faster responding during working memory (Enge et al., 2011) and attention (Fan et al., 2003). However, the combination of our neurophysiological EEG results with the fact that the N2 ERP is smaller during working memory updating (Enge et al., 2011) suggest that individuals with the High MAO-A phenotype may have less control, though more testing will be needed to determine whether the High MAO-A group is engaged in less cognitive control or is processing information more efficiently.

Interestingly, during source memory the left parietal old/new effect was present in the High MAO-A group, even though it was not present during item memory (Figure 6). The only difference between the item memory and source memory tasks were the instructions and choice options given to participants during the test phase as the same encoding instructions were given for both the item and source memory study phases. During the source memory retrieval test, participants were specifically instructed to remember the task used during the study phase whereas the item memory task only instructed participants to indicate whether the test stimulus was old or new. Therefore, participants in the source memory task knew they needed to retrieve a contextual detail in order to successfully accomplish the task. This expectation may explain why the left parietal old/new effect is present in High MAO-A individuals during source memory and was not present during item memory.

### Limitations

Studies examining the relationship between SNP’s and behavioral changes in cognitive abilities have small effect sizes (Chabris et al., 2012), requiring large participant numbers. Our current study uses a relatively small sample (*n* = 59) which may explain the lack of differences in proportion of correct responses and source misattribution differences between the High and Low MAO-A groups. However, our study relies on examining how ERP correlates of recognition memory change and the effect size observed for the left parietal old/new effect in the Low MAO-A group is moderate (Cohen’s *d* = 0.41). Additionally, the effect size observed for response sensitivity differences between groups was moderate (Cohen’s *d* = 0.57) during source memory and large (Cohen’s *d* = 0.71) during item memory. The medium to large effect sizes combined with the finding that on two different test days for two different memory tasks, item and source response sensitivity was lower for High MAO-A participants gives us confidence that *MAO-A* phenotype influences response selectivity and the left parietal old/new effect.

## Conclusion

One potential path to understanding individual differences in cognitive function is to examine how different genetic phenotypes impact the neurophysiology supporting cognition. In our study, we examined how phenotypes of the *MAO-A* gene impact EEG correlates of both item and source recognition memory. Pre-clinical studies that either knock-out the *MAO-A* gene (Kim et al., 1997; Singh et al., 2013) or inhibit MAO-A enzymatic activity (Steckler et al., 2001; Wong et al., 2010; Simpson et al., 2012) lead to increased memory performance. In contrast, genetic studies in humans indicate that individuals with presumably High MAO-A activity are faster at working memory (Enge et al., 2011), attentional control (Fossella et al., 2002; Fan et al., 2003) and spatial navigation (Mueller et al., 2014) tasks. Our behavioral and ERP results shed some light on the apparent contradiction in the pre-clinical and human studies. Individuals in the Low MAO-A group, which would be more similar to knock-out of the *MAO-A* gene or inhibition of MAO-A activity, have higher response sensitivity scores and a left parietal old/new effect, suggesting the presence of a stronger memory signal. In animals, this stronger memory signal resulting from lower MAO-A enzymatic activity may lead to increased performance. In contrast, individuals with the *MAO-A* phenotype leading to high levels of MAO-A enzymatic activity may have increased physiological noise and may response more carelessly leading to a decreased ability to differentiate old and new items.

## Author Contributions

RSR analyzed the behavioral and EEG data, interpreted the data and wrote the manuscript. AS conducted all genetic testing and edited the manuscript. TC helped conceptualize the project and edited the manuscript. EN conceptualized the project, collected the data and edited the manuscript.

## Conflict of Interest Statement

The authors declare that the research was conducted in the absence of any commercial or financial relationships that could be construed as a potential conflict of interest.

## References

[B1] Ally B. A. Budson A. E. (2007). The worth of pictures: using high density event-related potentials to understand the memorial power of pictures and the dynamics of recognition memory. Neuroimage 35, 378–395. 10.1016/j.neuroimage.2006.11.023 17207639 PMC1852523

[B2] Ally B. A. McKeever J. D. Waring J. D. Budson A. E. (2009). Preserved frontal memorial processing for pictures in patients with mild cognitive impairment. Neuropsychologia 47, 2044–2055. 10.1016/j.neuropsychologia.2009.03.015 19467355 PMC2724267

[B3] Barnett J. H. Xu K. Heron J. Goldman D. Jones P. B. (2011). Cognitive effects of genetic variation in monoamine neurotransmitter systems: a population-based study of *COMT*, *MAOA*, and *5HTTLPR*. Am. J. Med. Genet. B Neuropsychiatr. Genet. 156, 158–167. 10.1002/ajmg.b.31150 21302344 PMC3494973

[B4] Cases O. Seif I. Grimsby J. Gaspar P. Chen K. Pournin S.. (1995). Aggressive behavior and altered amounts of brain serotonin and norepinephrine in mice lacking MAOA. Science 268, 1763–1766. 10.1126/science.7792602 7792602 PMC2844866

[B5] Chabris C. F. Hebert B. M. Benjamin D. J. Beauchamp J. Cesarini D. van der Loos M.. (2012). Most reported genetic associations with general intelligence are probably false positives. Psychol. Sci. 23, 1314–1323. 10.1177/0956797611435528 23012269 PMC3498585

[B6] Colzato L. S. de Rover M. van den Wildenberg W. P. M. Nieuwenhuis S. (2013a). Genetic marker of norepinephrine synthesis predicts individual differences in post-error slowing: a pilot study. Neuropsychologia 51, 2600–2604. 10.1016/j.neuropsychologia.2013.07.026 23962674

[B7] Colzato L. S. Zmigrod A. Hommel B. (2013b). Dopamine, norepinephrine, and the management of sensorimotor bindings: individual differences in upating of stimulus-response episodes are predicted by DAT1, but not DBH5’-ins/del. Exp. Brain Res. 228, 213–220. 10.1007/s00221-013-3553-x 23681294

[B8] Curran T. (2000). Brain potentials of recollection and familiarity. Mem. Cognit. 28, 923–938. 10.3758/bf03209340 11105518

[B9] Curran T. Hancock J. (2007). The FN400 indexes familiarity-based recognition of faces. Neuroimage 36, 464–471. 10.1016/j.neuroimage.2006.12.016 17258471 PMC1948028

[B10] Curran T. Schacter D. L. Johnson M. K. Spinks R. (2001). Brain potentials reflect behavioral differences in true and false recognition. J. Cogn. Neurosci. 13, 201–216. 10.1162/089892901564261 11244546

[B11] Dannlowski U. Ohrmann P. Konrad C. Domschke K. Bauer J. Kugel H.. (2009). Reduced amygdala-prefrontal coupling in major depression: association with MAOA genotype and illness severity. Int. J. Neuropharmacol. 12, 11–22. 10.1017/s1461145708008973 18544183

[B12] Davachi L. Mitchell J. P. Wagner A. D. (2003). Multiple routes to memory: distinct medial temporal lobe processes build item and source memories. Proc. Natl. Acad. Sci. U S A 100, 2157–2162. 10.1073/pnas.0337195100 12578977 PMC149975

[B13] Delorme A. Makeig S. (2004). EEGLAB: an open source toolbox for analysis of single-trial EEG dynamics including independent component analysis. J. Neurosci. Methods 134, 9–21. 10.1016/j.jneumeth.2003.10.009 15102499

[B14] Depue B. E. Ketz N. Mollison M. V. Nyhus E. Banich M. T. Curran T. (2013). ERPs and neural oscillations during volitional suppression of memory retrieval. J. Cogn. Neurosci. 25, 1624–1633. 10.1162/jocn_a_00418 23647560

[B15] Donaldson D. I. Rugg M. D. (1998). Recognition memory for new associations: electrophysiological evidence for the role of recollection. Neuropsychologia 36, 377–395. 10.1016/s0028-3932(97)00143-7 9699947

[B16] Donaldson D. I. Rugg M. D. (1999). Event-related potential studies of associative recognition and recall: electrophysiological evidence for context dependent retrieval processes. Cogn. Brain Res. 8, 1–16. 10.1016/s0926-6410(98)00051-2 10216269

[B17] Eichenbaum H. Yonelinas A. R. Ranganath C. (2007). The medial temporal lobe and recognition memory. Annu. Rev. Neurosci. 30, 123–152. 10.1146/annurev.neuro.30.051606.094328 17417939 PMC2064941

[B18] Enge S. Fleischhauer M. Lesch K. P. Reif A. Strobel A. (2011). Serotonergic modulation in executive functioning: linking genetic variations to working memory performance. Neuropsychologia 49, 3776–3785. 10.1016/j.neuropsychologia.2011.09.038 21983350

[B19] Evans L. H. Wilding E. L. Hibbs C. S. Herron J. E. (2010). An electrophysiological study of boundary conditions for control of recollection in the exclusion task. Brain Res. 1324, 43–53. 10.1016/j.brainres.2010.02.010 20153299

[B20] Fan J. Fossella J. Sommer T. Wu Y. Posner M. I. (2003). Mapping the genetic variation of executive function onto brain activity. Proc. Natl. Acad. Sci. U S A 100, 7406–7411. 10.1073/pnas.0732088100 12773616 PMC165888

[B21] Folstein J. R. Van Petten C. (2008). Influence of cognitive control and mismatch on the N2 component of the ERP: a review. Psychophysiology 45, 152–170. 10.1111/j.1469-8986.2007.00602.x 17850238 PMC2365910

[B22] Fossella J. Sommer T. Fan J. Wu Y. Swanson J. M. Pfaff D. W.. (2002). Assessing the molecular genetics of attention networks. BMC Neurosci. 3:14. 10.1186/1471-2202-3-14 12366871 PMC130047

[B23] Friedman N. P. Miyake A. Young S. E. DeFries J. C. Corley R. P. Hewitt J. K. (2008). Individual differences in executive functions are almost entirely genetic in origin. J. Exp. Psychol. Gen. 137, 201–225. 10.1037/0096-3445.137.2.201 18473654 PMC2762790

[B25] Hayama H. R. Johnson J. D. Rugg M. D. (2008). The relationship between the right frontal old/new ERP effect and post-retrieval monitoring: specific or non-specific? Neuropsychologia 46, 1211–1223. 10.1016/j.neuropsychologia.2007.11.021 18234241 PMC2441597

[B24] Hayama H. R. Rugg M. D. (2009). Right dorsolateral prefrontal cortex is engaged during post-retrieval processing of both episodic and semantic information. Neuropsychologia 47, 2409–2416. 10.1016/j.neuropsychologia.2009.04.010 19383503 PMC2712584

[B26] Johansson M. Mecklinger A. (2003). The late posterior negativity in ERP studies of episodic memory: action monitoring and retrieval of attribute conjunctions. Biol. Psychol. 64, 91–117. 10.1016/s0301-0511(03)00104-2 14602357

[B27] Kahn I. Davachi L. Wagner A. D. (2004). Functional-neuroanatomic correlates of recollection: implications for models of recognition memory. J. Neurosci. 24, 4172–4180. 10.1523/JNEUROSCI.0624-04.2004 15115812 PMC6729281

[B28] Kim J. J. Shih J. C. Chen K. Chen L. Bao S. Maren S.. (1997). Selective enhancement of emotional, but not motor, learning in monoamine oxidase A-deficient mice. Proc. Natl. Acad. Sci. U S A 94, 5929–5933. 10.1073/pnas.94.11.5929 9159177 PMC20883

[B29] Kropotov J. D. Ponomarev V. A. Pronina M. Jäncke L. (2017). Functional indexes of reactive cognitive control: ERPs in cued go/no-go tasks. Psychophysiology 54, 1899–1915. 10.1111/psyp.12960 28771747

[B30] Kucera H. Francis W. (1967). Computational Analysis of Present-Day American English. Providence: Brown University Press.

[B31] Leynes P. A. Kakadia B. (2013). Variations in retrieval monitoring during action memory judgments: evidence form event-related potentials (ERPs). Int. J. Psychophysiol. 87, 189–199. 10.1016/j.ijpsycho.2013.01.004 23313607

[B32] Leynes P. A. Phillips M. C. (2008). Event-related potential (ERP) evidence for varied recollection during source monitoring. J. Exp. Psychol. Learn. Mem. Cogn. 34, 741–751. 10.1037/0278-7393.34.4.741 18605865

[B33] Lopez-Calderon J. Luck S. J. (2014). ERPLAB: an open-source toolbox for the analysis of event-related potentials. Front. Hum. Neurosci. 8:213. 10.3389/fnhum.2014.00213 24782741 PMC3995046

[B34] Markett S. Montag C. Walter N. T. Plieger T. Reuter M. (2011). On the molecular genetics of flexibility: the case of task-switching, inhibitory control and genetic variants. Cogn. Affect. Behav. Neurosci. 11, 644–651. 10.3758/s13415-011-0058-6 21994116

[B35] Mecklinger A. (2006). Electrophysiological measures of familiarity memory. Clin. EEG Neurosci. 37, 292–299. 10.1177/155005940603700406 17073167

[B36] Mecklinger A. Johansson M. Parra M. Hanslmayr S. (2007). Source-retrieval requirements influence late ERP and EEG memory effects. Brain Res. 1172, 110–123. 10.1016/j.brainres.2007.07.070 17822684

[B37] Mecklinger A. Rosburg T. Johannson M. (2016). Reconstructing the past: the late posterior negativity (LPN) in episodic memory studies. Neurosci. Biobehav. Rev. 68, 621–638. 10.1016/j.neubiorev.2016.06.024 27365154

[B38] Medrano P. Nyhus E. Smolen A. Curran T. Ross R. S. (2017). Individual differences in EEG correlates of recognition memory due to DAT polymorphisms. Brain Behav. 7:e00870 10.1002/brb3.870 29299388 PMC5745248

[B39] Mueller S. C. Cornwell B. R. Grillon C. MacIntyre J. Gorodetsky E. Goldman D.. (2014). Evidence of MAOA genotype involvement in spatial ability in males. Behav. Brain Res. 267, 106–110. 10.1016/j.bbr.2014.03.025 24671068 PMC4548810

[B40] Norman K. A. Tepe K. Nyhus E. Curran T. (2008). Event-related potential correlates of interference effects on recognition memory. Psychon. Bull. Rev. 15, 36–43. 10.3758/pbr.15.1.36 18605477

[B41] Nyhus E. Curran T. (2009). Semantic and perceptual effects on recognition memory: evidence from ERP. Brain Res. 1283, 102–114. 10.1016/j.brainres.2009.05.091 19505439 PMC2748123

[B42] Pires L. Leitão J. Guerrini C. Simões M. R. (2014). Event-related brain potentials in the study of inhibition: cognitive control, source localization and age-related modulations. Neuropsychol. Rev. 24, 461–490. 10.1007/s11065-014-9275-4 25407470

[B43] Ranganath C. Paller K. A. (2000). Neural correlates of memory retrieval and evaluation. Cogn. Brain Res. 9, 209–222. 10.1016/s0926-6410(99)00048-8 10729705

[B44] Rosburg T. Johansson M. Mecklinger A. (2013). Strategic retrieval and retrieval orientation in reality monitoring studies by event-related potentials (ERPs). Neuropsychologia 51, 557–571. 10.1016/j.neuropsychologia.2012.11.014 23168131

[B45] Rosburg T. Mecklinger A. Johansson M. (2011). Strategic retrieval in a reality monitoring task. Neuropsychologia 49, 2957–2969. 10.1016/j.neuropsychologia.2011.07.002 21763332

[B46] Ross R. S. Medrano P. Boyle K. Smolen A. Curran T. Nyhus E. (2015). Genetic variation in the serotonin transporter gene influences the ERP old/new effect during recognition memory. Neuropsychologia 78, 95–107. 10.1016/j.neuropsychologia.2015.09.028 26423665 PMC4744468

[B47] Rugg M. D. Curran T. (2007). Event-related potentials and recognition memory. Trends Cogn. Sci. 11, 251–257. 10.1016/j.tics.2007.04.004 17481940

[B48] Rugg M. D. Henson R. N. A. Robb W. G. K. (2003). Neural correlates of retrieval processing in the prefrontal cortex during recognition and exclusion tasks. Neuropsychologia 41, 40–52. 10.1016/s0028-3932(02)00129-x 12427564

[B49] Rugg M. D. Mark R. E. Walla P. Schloerscheidt A. M. Birch C. S. Allan K. (1998). Dissociation of the neural correlates of implicit and explicit memory. Nature 392, 595–598. 10.1038/33396 9560154

[B50] Sabol S. Z. Hu S. Hamer D. (1998). A functional polymorphism in the monamine oxidase A gene promoter. Hum. Genet. 103, 273–279. 10.1007/s004390050816 9799080

[B51] Saura J. Kettler R. Da Prada M. Richards J. G. (1992). Quantitative enzyme radioautography with 3H-Ro41–1049 and 3H-Ro19–6327 *in vitro*: localization and abundance of MAO-A and MAO-B in rat CNS, peripheral organs and human brain. J. Neurosci. 12, 1977–1999. 1578281 10.1523/JNEUROSCI.12-05-01977.1992 PMC6575899

[B52] Simpson S. M. Hickey A. J. Baker G. B. Reynolds J. N. Beninger R. J. (2012). The antidepressant phenelzine enhances memory in the double Y-maze and increases GABA levels in the hippocampus and frontal cortex of rats. Pharmacol. Biochem. Behav. 102, 109–117. 10.1016/j.pbb.2012.03.027 22503968

[B53] Singh C. Bortolato M. Bali N. Godar S. C. Scott A. L. Chen K.. (2013). Cognitive abnormalities and hippocampal alterations in monoamine oxidase A and B knockout mice. Proc. Natl. Acad. Sci. U S A 110, 12816–12821. 10.1073/pnas.1308037110 23858446 PMC3732950

[B54] Srinivasan R. Nunez P. L. Tucker D. M. Silberstein R. B. Cadusch P. J. (1996). Spatial sampling and filtering of EEG with spline laplacians to estimate cortical potentials. Brain Topogr. 8, 355–366. 10.1007/bf01186911 8813415

[B55] Steckler T. Rammes G. Sauvage M. van Gaalen M. M. Weis C. Zieglgänsberger W.. (2001). Effects of the monoamine oxidase A inhibitor moclobemide on hippocampal plasticity in GR-impaired transgenic mice. J. Psychiatr. Res. 35, 29–42. 10.1016/s0022-3956(00)00040-6 11287054

[B56] Stelzel C. Basten U. Montag C. Reuter M. Fiebach C. J. (2010). Frontostriatal involvement in task switching depends on genetic differences in D2 receptor density. J. Neurosci. 30, 14205–14212. 10.1523/jneurosci.1062-10.2010 20962241 PMC6634769

[B57] Tadic A. Rujescu D. Szegedi A. Giegling I. Singer P. Möller H. J.. (2003). Association of a MAOA gene variant with generalized anxiety disorder, but not with panic disorder or major depression. Am. J. Med. Genet. B Neuropsychiatr. Genet. 117B, 1–6. 10.1002/ajmg.b.10013 12555227

[B58] van Holstein M. Aarts E. van der Schaaf M. E. Geurts D. E. M. Verkes R. J. Franke B.. (2011). Human cognitive flexibility depends on dopamine D2 receptor signaling. Psychopharmacology 218, 567–578. 10.1007/s00213-011-2340-2 21611724 PMC3210362

[B59] Verdejo-García A. Albein-Urios N. Molina E. Ching-López A. Martínez-González J. M. Gutiérrez B. (2013). A MAOA gene*cocaine severity interaction on impulsivity and neurophychological measures of orbitofrontal dysfunction: preliminary results. Drug Alcohol Depend. 133, 287–290. 10.1016/j.drugalcdep.2013.04.031 23755928

[B60] Voss J. L. Lucas H. D. Paller K. A. (2012). More than a feeling: pervasive influences of memory without awareness of retrieval. Cogn. Neurosci. 3, 193–207. 10.1080/17588928.2012.674935 24171735 PMC4385384

[B61] Werkle-Bergner M. Mecklinger A. Kray J. Meyer P. Düzel E. (2005). The control of memory retrieval: insights from event-related potentials. Cogn. Brain Res. 24, 599–614. 10.1016/j.cogbrainres.2005.03.011 16099369

[B62] Westlund K. N. Denney R. M. Rose R. M. Abell C. W. (1988). Localization of distinct monoamine oxidase A and monoamine oxidase B cell populations in human brainstem. Neuroscience 25, 439–456. 10.1016/0306-4522(88)90250-3 3399053

[B64] Wilding E. L. Fraser C. S. Herron J. E. (2005). Indexing strategic retrieval of colour information with event related potentials. Cogn. Brain Res. 25, 19–32. 10.1016/j.cogbrainres.2005.04.012 15923113

[B63] Wilding E. L. Rugg M. D. (1996). An event-related potential study of recognition memory with and without retrieval of source. Brain 119, 889–905. 10.1093/brain/119.3.889 8673500

[B65] Willoughby J. Glover V. Sandler M. (1988). Histochemical localization of monoamine oxidase A and B in rat brain. J. Neural. Transm. 74, 29–42. 3171572 10.1007/BF01243573

[B66] Wong F. K. Lee S. H. Atcha A. Ong A. B. Pemberton D. J. Chen W. S. (2010). Rasagiline improves learning and memory in young healthy rats. Behav. Pharmacol. 21, 278–282. 10.1097/fbp.0b013e32833aec02 20520531

